# Provider-Identified Deprescribing Facilitators and Strategies for Older Adults in Primary Care: A Team Alice Study

**DOI:** 10.1093/geroni/igaa057.1209

**Published:** 2020-12-16

**Authors:** Laura Brady, Susan LaValley, Molly Ranahan, Collin Clark, Scott Monte, David Jacobs, Robert Wahler, Ranjit Singh

**Affiliations:** 1 SUNY University at Buffalo, Buffalo, New York, United States; 2 University at Buffalo, Buffalo, New York, United States; 3 Erie County Medical Center, Buffalo, New York, United States; 4 University at Buffalo School of Pharmacy and Pharmaceutical Sciences, Buffalo, New York, United States; 5 University at Buffalo School of Pharmacy, Orchard Park, New York, United States; 6 University at Buffalo, Buffalo, United States; 7 State Univ. of New York at Buffalo, Buffalo, New York, United States

## Abstract

Potentially inappropriate medications (PIMs) may harm adults over the age of 65, yet PIMs are prescribed at high rates. The process of deprescribing PIMs is challenging in the primary care setting, particularly among older adult patients with multiple chronic conditions. While barriers to deprescribing are well known, less data are available on the facilitators and strategies that primary care providers consider key to successful deprescribing. This study examines providers’ perceptions and attitudes of deprescribing to identify individual and systems-level facilitators and strategies for successful deprescribing. Data were collected through semi-structured interviews with 20 providers recruited from primary care practices located in Western New York. Rapid thematic analysis was used to identify the facilitators and strategies providers perceived as important to successful deprescribing. Facilitators included providers adapting their approach to deprescribing PIMs based on their knowledge of the patient. Providers’ own characteristics were also important, as were those of their organization, including whether a clinical pharmacist was available to consult. Strategies for deprescribing were patient-focused (e.g., adapting to patient’s lifestyle), process-focused (e.g., patient education on polypharmacy), and medication-focused (e.g., tapering). It is clear that many of the primary care providers who treat a larger number of older adults are aware of the importance of deprescribing PIMs. However, deprescribing in a busy primary-care setting is challenging. Findings detailing providers’ perceptions of facilitators and strategies for deprescribing can guide future interventions and target support to reduce the risk of harm from PIMs in older adults.

